# Visual Motor and Perceptual Task Performance in Astigmatic Students

**DOI:** 10.1155/2017/6460281

**Published:** 2017-02-15

**Authors:** Erin M. Harvey, J. Daniel Twelker, Joseph M. Miller, Tina K. Leonard-Green, Kathleen M. Mohan, Amy L. Davis, Irene Campus

**Affiliations:** ^1^Department of Ophthalmology and Vision Science, The University of Arizona, Tucson, AZ, USA; ^2^Mel and Enid Zuckerman College of Public Health, The University of Arizona, Tucson, AZ, USA; ^3^College of Optical Sciences, The University of Arizona, Tucson, AZ, USA

## Abstract

*Purpose*. To determine if spectacle corrected and uncorrected astigmats show reduced performance on visual motor and perceptual tasks.* Methods*. Third through 8th grade students were assigned to the low refractive error control group (astigmatism < 1.00 D, myopia < 0.75 D, hyperopia < 2.50 D, and anisometropia < 1.50 D) or bilateral astigmatism group (right and left eye ≥ 1.00 D) based on cycloplegic refraction. Students completed the Beery-Buktenica Developmental Test of Visual Motor Integration (VMI) and Visual Perception (VMIp). Astigmats were randomly assigned to testing with/without correction and control group was tested uncorrected. Analyses compared VMI and VMIp scores for corrected and uncorrected astigmats to the control group.* Results*. The sample included 333 students (control group 170, astigmats tested with correction 75, and astigmats tested uncorrected 88). Mean VMI score in corrected astigmats did not differ from the control group (*p* = 0.829). Uncorrected astigmats had lower VMI scores than the control group (*p* = 0.038) and corrected astigmats (*p* = 0.007). Mean VMIp scores for uncorrected (*p* = 0.209) and corrected astigmats (*p* = 0.124) did not differ from the control group. Uncorrected astigmats had lower mean scores than the corrected astigmats (*p* = 0.003).* Conclusions*. Uncorrected astigmatism influences visual motor and perceptual task performance. Previously spectacle treated astigmats do not show developmental deficits on visual motor or perceptual tasks when tested with correction.

## 1. Introduction

Astigmatic blur in early childhood can result in reduced visual performance (e.g., poor visual acuity when spectacles are not worn) as well as poor visual development (e.g., poor visual acuity that persists when spectacles are worn (astigmatism-related amblyopia)) [1]. In addition, several studies have suggested that astigmatism may influence other aspects of childhood development and performance of more complex tasks [2–4].

There is little data in the literature on the effects of astigmatism on performance of visual motor or perceptual tasks. However, there is some evidence that ametropia can influence visual motor performance. Atkinson and her colleagues compared performance on the Movement Assessment Battery for Children (Movement ABC) for children who were emmetropic at age 9 months and children who were hyperopic (≥3.50 D, some were also astigmatic) at age 9 months [5, 6]. Significantly reduced performance was observed for children in the hyperopic group when tested at ages 3.5 and 5.5 years, even though children who remained significantly hyperopic wore spectacles for testing. This effect persisted when amblyopic, strabismic, and preterm children were excluded. Roch-Levecq et al. [7] reported that uncorrected bilaterally ametropic (hyperopic and/or astigmatic) preschoolers had reduced scores on the Beery-Buktenica Developmental Test of Visual Motor Integration (VMI) [8] compared to emmetropic preschoolers. However, when tested with spectacle correction the bilaterally ametropic preschoolers showed performance comparable to the emmetropic control group. Orlansky et al. reported lower scores on several measures of academic readiness in uncorrected astigmatic preschoolers compared to their nonastigmatic peers [3]. However, there was no significant difference between uncorrected astigmatic and nonastigmatic preschoolers on the measure that most closely reflected visual motor performance and fine motor skills, perhaps due in part to the fact that some of their astigmatic students had little astigmatism (the cutoff for inclusion in the astigmatism group was ≥0.50 D). In summary, these studies provide inconsistent results for both corrected and uncorrected ametropic preschoolers compared to emmetropic preschoolers. Differences in findings are likely due, in part, to differences in the study tasks. In assessment of uncorrected ametropes, one study used the VMI [7], which focuses specifically on a near visual motor task, and the other used a variety of measures of academic readiness, one of which measured fine motor skills [3]. In assessment of corrected ametropes, one study used the Movement ABC, which includes a variety of motor and visual motor tasks [5, 6], and the other used the VMI [7].

In the present study we test two hypotheses. The first hypothesis is that blur or difficulty in focusing during near tasks due to uncorrected astigmatism reduces visual motor and perceptual performance. While a reduction in performance in uncorrected astigmats, if observed, could be due to difficulties introduced by astigmatic blur, we must rule out the possibility that some or all of any reduced performance in uncorrected astigmats is due to poor visual motor or perceptual development resulting from persistent blur during early childhood development or to astigmatism-related amblyopia. The second hypothesis, aimed at distinguishing between effects of blur and effects of poor visual motor or perceptual development, is that astigmats will show reduced visual motor and perceptual performance even when wearing spectacle correction. To test these hypotheses, we conducted visual motor and perceptual testing on two groups of bilateral astigmatic students: one group tested with spectacle correction and one group tested without correction. Results were compared to a control group of students with low refractive error from the same cohort.

## 2. Methods

### 2.1. Subjects

Participants were 3rd through 8th grade students who attended schools on the Tohono O'odham reservation during the 2013/14 school year. All students in grades 3–8 were invited to participate. Previous research has documented a high prevalence of with-the-rule (WTR) astigmatism in Tohono O'odham school-age children [9]. Previous research documenting a high prevalence of astigmatism in Tohono O'odham infants and toddlers [10] and little change in astigmatism in Tohono O'odham children through the preschool and grade school years [11, 12] suggest that astigmatic Tohono O'odham school-age children are likely to have had astigmatism from a young age.

This study complied with the Declaration of Helsinki and was approved by the Tohono O'odham Nation and the Institutional Review Board of the University of Arizona. Written informed consent was obtained from parents and written assent was obtained from students prior to testing.

### 2.2. Procedures

#### 2.2.1. Eye Examination

Uncorrected binocular recognition acuity was measured at a distance of 3 meters using logMAR letter acuity charts (ETDRS charts, Precision Vision, LaSalle, IL). Final acuity estimate was the smallest LogMAR line on which the student was able to correctly identify at least 3 of 5 letters.

After acuity testing, one drop of 0.5% proparacaine followed by a drop of 1% tropicamide and one drop of 1% cyclopentolate was administered. At least 30 minutes later, cycloplegic autorefraction was performed with the Retinomax KPlus2 (Nikon, Inc. Melville NY). The autorefraction result was adjusted as needed through subjective refinement to determine a best estimate of refractive error. Spectacles were prescribed for students who met any of the following criteria: astigmatism ≥ 1.00 D in either eye, myopia ≥ 0.75 D on any meridian in either eye, hyperopia ≥ 2.50 D on any meridian in either eye, and anisometropia ≥ 1.50 D spherical equivalent (SE). The sphere correction was reduced by 1/3 or by 1.00 D (whichever was greater) for students with hyperopia. The full correction was prescribed for astigmatism and myopia.

On a subsequent day (once spectacles became available), binocular best-corrected visual acuity was measured using the same method used for uncorrected acuity (summarized above). Astigmatic students wore their spectacle correction, and students who did not meet prescribing criteria wore a pair of spectacles that closely matched their right and left eye refractive error correction (within 0.50 vector difference in diopters) [13].

#### 2.2.2. Visual Motor and Perception Testing

Students completed the Full Form Beery-Buktenica Developmental Test of Visual Motor Integration 6th Edition (VMI) and the Beery VMI Developmental Test of Visual Perception 6th Edition (VMIp) (Pearson Clinical Assessment, Bloomington, MN) [8]. The VMI was chosen because it is widely used and validated and has been shown to be sensitive enough to detect deficits due to hyperopic blur [7]. The VMI assessment consists of a series of line drawings of geometric forms (ranging in difficulty from a single line or circle to “three-dimensional” line drawings) that are to be copied as accurately as possible. The VMI stimuli are high contrast and range in size from approximately 4.9 to 7.4 cm (maximum width). On the VMIp, students are shown a standard form with several similar forms below it. The task is for the student to identify the form that is identical to the standard. Forms on the VMIp are also high contrast but are smaller in size than the forms on the VMI: individual standard forms range in size from approximately 1 to 2 cm. The VMI and VMIp utilize the same 24 standard forms.

The VMI was always administered first, followed by the VMIp. Astigmatic students completed VMI and VMIp testing at least 2 weeks after spectacles were dispensed. Students who were prescribed spectacles were randomly assigned to complete the VMI and VMIp tests either with or without spectacle correction. Students in the control group completed the VMI and VMIp tests without correction.

Testing was conducted according to the VMI Manual [8]. Students were seated at a table and were given a pen and a test booklet open to the first page of test forms. Testing distance from the VMI booklets was not controlled although students were instructed to keep the book square in front of them. The tester instructed the student to copy each form in the space below it and gave an example using a form that is not included in the test. Students were to “copy the forms in order, do their best (even though some of them are hard, even for adults), and to remember that they cannot erase.” Once the students completed all forms, the tester collected the booklet and proceeded with VMIp testing, which was also conducted according to the VMI Manual [8]. For each item, students were shown a standard form with several forms below it and were asked to select the form that was identical to the standard. Students were encouraged to look carefully at each form before making their selection.

#### 2.2.3. Data Analysis

The VMI tests were scored by two research team members who strictly applied the objective criteria outlined in the Beery VMI Scoring Manual [8]. Scorers used rulers and protractors for scoring, as needed. For each form on the VMI, scores (1 if it met the scoring criteria and 0 if it did not) were compared across scorers. In instances where the scorers disagreed, a third researcher reviewed the form and made a final determination. The VMIp, which is a multiple choice assessment, was scored by one research team member and verified by another. The VMI and VMIp raw scores were converted to standardized scores (age based norms determined from a sample of 1,737 children) [8].

Students were assigned to the following groups based on their best estimate of refractive error: control group (students did not meet any criteria for glasses prescription: astigmatism in both eyes < 1.00 D, myopia < 0.75 D on any meridian in either eye, and hyperopia < 2.50 D on any meridian in either eye) or bilateral astigmatism (astigmatism in both eyes ≥ 1.00 D). Data from students with ocular abnormalities, anisometropia (>1.50 D SE or >1.50 D astigmatism), or refractive error that did not meet the criteria for either the control group or the bilateral astigmatism group were excluded from analyses.

Preliminary analyses compared three groups (control group, astigmats tested with correction, and astigmats tested without correction) on demographics (age, gender), vision status (uncorrected and best-corrected binocular distance acuity), and refractive error (spherical equivalent, astigmatism) to determine if the groups were comparable with respect to these variables.

Separate analyses were conducted for VMI and VMIp data. Analysis of Covariance (ANCOVA) was used to compare mean scores across groups while controlling for age. Post hoc analyses applied Bonferroni correction for multiple comparisons.

Secondary ANCOVA analyses were also conducted in which we controlled for visual acuity. In order for the visual acuity variable to be representative of acuity under the students' VMI/VMIp testing condition, a new acuity variable was created in which uncorrected acuity was used for the control group and for the astigmatic group who completed the VMI and VMIp without correction and best-corrected acuity was used for the astigmatic group who completed the VMI and VMIp with correction.

Additional ANCOVA analyses, including only data from astigmatic students tested without correction, were conducted to determine if astigmatism magnitude influenced VMI and VMIp scores. Astigmatic students were categorized as having moderate or high astigmatism. ANCOVA, including age as a covariate, compared mean scores for uncorrected astigmatic students with moderate (mean of right and left eye astigmatism 1.00 to < 3.00 D) versus high (mean of right and left eye astigmatism ≥ 3.00 D) astigmatism.

## 3. Results

### 3.1. Study Sample

A total of 333 students met inclusion criteria: 170 with low refractive error (control group) and 163 with bilateral astigmatism.  Table 1 compares characteristics of the three study groups: control group, astigmats who completed the VMI and VMIp without spectacle correction, and astigmats who completed the VMI and VMIp with spectacle correction. The three groups did not significantly differ on mean age or gender. The control group had significantly better uncorrected and corrected distance acuity than both of the astigmatic groups (ANOVA *p* values < 0.001, post hoc *p* values < 0.001). The two groups of astigmatic students did not significantly differ on mean uncorrected or corrected distance acuity. Mean spherical equivalent refractive error did not significantly differ across the three study groups for right (*p* = 0.263) or left eyes (*p* = 0.455), and the two groups of astigmatic students did not significantly differ on mean right (*p* = 0.649) or left eye (*p* = 0.498) astigmatism magnitude.

All but 5 of the 163 astigmatic students had participated in at least one previous study through which they were provided spectacles (first study prescription given an average of 5.37 years (range 0.87 to 9.73 years) prior to participation in the present study). Of the 5 astigmatic students who had not participated in previous studies, 4 reported previous spectacle wear (duration of previous wear not recorded) and one reported no previous spectacle wear (per student and parent report). Despite the high rate of previous wear (99%), only 49.7% (81/163) of astigmatic students arrived at the study eye examination with spectacles.

### 3.2. VMI Results

ANCOVA yielded a significant main effect of study group (control, uncorrected astigmats, and corrected astigmats, *p* = 0.006) and a significant effect of the covariate, age (*p* < 0.001), on VMI standardized scores. Post hoc analyses (using Bonferroni correction) indicated that the uncorrected astigmatic group had significantly lower mean VMI scores than both the control group (*p* = 0.038) and the corrected astigmatic group (*p* = 0.007). Results for the control group did not significantly differ from the corrected astigmatic group (*p* = 0.829).

The secondary ANCOVA analysis, which included age and visual acuity, yielded significant effects of age (*p* < 0.001) and acuity (*p* = 0.009), but the effect of group was not statistically significant (*p* = 0.196).

ANCOVA comparing mean VMI score for uncorrected moderate (*n* = 40) versus high astigmats (*n* = 48) yielded a significant effect of age (*p* = 0.003) but no significant effect of astigmatism magnitude (*p* = 0.872).

### 3.3. VMIp Results

ANCOVA yielded a significant main effect of study group (*p* = 0.004) and a significant effect of the covariate, age (*p* < 0.001), on VMIp standardized scores. Post hoc analyses indicated that the control group did not significantly differ from the uncorrected astigmatic group (*p* = 0.209) or the corrected astigmatic group (*p* = 0.124). The uncorrected astigmatic group had significantly lower mean VMIp scores than the corrected astigmatic group (*p* = 0.003).

The secondary ANCOVA analysis, which included age and visual acuity covariates, yielded a significant effect of group (*p* = 0.016) as well as significant effects of age (*p* < 0.001) and acuity (*p* = 0.002). Post hoc analyses indicated that the corrected astigmatic group performed significantly* better* than the control group (*p* = 0.013). No other post hoc comparisons were significant.

ANCOVA comparing mean VMIp score for uncorrected moderate versus high astigmats yielded a significant effect of age (*p* < 0.001) but no significant effect of astigmatism magnitude (*p* = 0.396).

## 4. Discussion

The hypothesis that astigmatic students may show reduced visual motor performance when uncorrected was supported by the VMI data. Uncorrected astigmatic students performed more poorly on the VMI than students in the control and corrected astigmatism groups. When we controlled for visual acuity in the secondary analysis, the differences across groups were no longer significant. These results suggest that astigmatic blur influenced performance on the VMI, even though the VMI stimuli are high contrast and relatively large (4.9 to 7.4 cm in width) and viewing distance was not controlled during task performance.

Performance on the VMIp did not significantly differ across control and uncorrected astigmatism groups. However, corrected astigmats performed significantly better than uncorrected astigmats. When analysis controlled for visual acuity, the corrected and uncorrected astigmats no longer significantly differed. These results suggest that the improved vision when spectacles are worn resulted in improved performance on the VMIp. The VMIp forms are relatively small in size (1 to 2 cm) and the task requires students to detect subtle differences in small features across similar forms. It is not clear if the same effect would be observed for larger stimuli (e.g., stimuli comparable in size to the forms presented on the VMI test).

From a clinical perspective, determining the magnitude of uncorrected astigmatism sufficient to significantly reduce VMI or VMIp is important, as it could influence spectacle prescribing recommendations. For astigmatic students tested without correction, performance on the VMI and the VMIp did not significantly vary by magnitude of uncorrected astigmatism. Therefore, while the present study suggested that uncorrected astigmatism ≥ 1.00 D may impact visual motor or perceptual performance, results from the present study did not yield more specific information regarding the level of astigmatism at which students are at increased risk for reduced VMI or VMIp performance.

Our second hypothesis that* corrected* astigmats may show reduced visual motor or perceptual performance was not supported by the data. Corrected astigmatic students performed comparably to students in the control group on both the VMI and the VMIp. These data suggest either that uncorrected astigmatism in early childhood does not result in poor visual motor or perceptual development or that correction of astigmatism over time can alleviate any developmental deficits and lead to normal performance on visual motor or perceptual tasks. However, our data cannot distinguish between these two conclusions. Astigmats had significantly reduced best-corrected acuity compared to students in the control group, but deficits were mild: on average, corrected acuity in the control group was 20/16 compared to 20/20 in the astigmatic groups. Such small deficits could be attributable to amblyopia, spectacle lens distortion, or residual uncorrected refractive error and may not be sufficient in magnitude to influence VMI or VMIp performance. Almost all of the astigmatic students had a history of spectacle wear prior to the study exam and our acuity data suggest that amblyopia may have been successfully treated in many of the astigmatic students. It is not clear if the students in our study had reduced visual motor performance prior to initiation of spectacle treatment. Previously untreated astigmats of the same age may show deficits in visual motor or perceptual skills.

In summary, the finding of reduced visual motor performance in uncorrected astigmats suggests that response to high contrast above acuity threshold visual motor stimuli is influenced by astigmatic blur. The fact that reduced visual motor performance was not observed in corrected astigmats rules out the possibility that reduced performance when uncorrected was due to poor visual motor development. Our results are consistent with the finding of Roch-Levecq et al. [7], who observed reduced performance on the VMI in uncorrected ametropic (hyperopic and/or astigmatic) preschoolers compared to an emmetropic control group, but comparable performance in corrected ametropes (tested after 6 weeks of spectacle wear) and emmetropes. Perceptual performance in uncorrected astigmats did not differ from the control group but was significantly reduced compared to corrected astigmats, suggesting that spectacles also improved performance on the VMIp task. However, unlike the VMI which included relatively large stimuli, reduced performance on the VMIp in uncorrected astigmats may be due in part to poor visual acuity as the critical features of the VMIp stimuli that are necessary for performing the task may have been below acuity threshold for some uncorrected astigmats.

**Figure 1:**
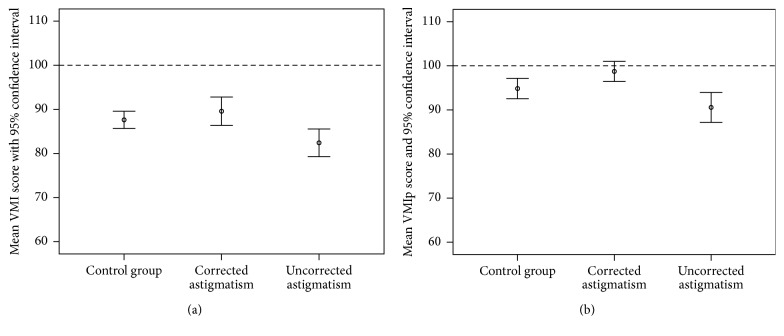
Mean VMI (a) and VMIp (b) standardized scores for students with low refractive error (control group) tested without correction, bilateral astigmats tested with correction, and bilateral astigmats tested without correction. Error bars represent the 95% confidence interval for the mean. Dashed line represents the average score for standardized norms [8].

This study has several strengths. First, we include a large sample of bilateral astigmatic students and a control group of students with low refractive error from the same cohort tested in the same manner. Second, preliminary analyses indicated that our three study groups were comparable in terms of gender, mean age, and mean spherical equivalent refractive error, although the range of spherical equivalent refractive errors was much wider in the astigmatic groups compared to the control group. The primary limitations of the study relate to the generalizability of the findings. All participants were Tohono O'odham and previous research has produced conflicting results regarding racial, cultural, or ethnic differences in VMI scores [8, 14–19]. Students performed below the average standardized score of 100 (see Figures 1(a) and 1(b)). This could be due to our strict use of objective VMI scoring criteria, or it could reflect a cultural bias in the VMI and VMIp assessments. However, we did not assess performance of astigmats in comparison to standardized norms but rather assessed performance relative to a control group from the same cohort, thus reducing concern that any cultural bias in the VMI or VMIp influenced the primary study findings. An additional limitation regarding generalizability relates to the fact that all of the astigmats in the present study were with-the-rule astigmats, and it is not clear if the same results would be observed in children with against-the-rule or oblique astigmatism. Finally, almost all astigmats in this study had previous spectacle treatment. As a result, we cannot determine from this study if students with previously uncorrected astigmatism and/or students with astigmatism-related amblyopia might show reduced performance on visual motor or perceptual tasks when corrected. We can conclude, however, that, with correction, spectacle treated astigmatic students can perform comparably to their nonastigmatic peers.

Research on the effects of refractive errors typically focuses on traditional measures of vision, such as visual acuity, contrast sensitivity, and stereo acuity. The results of the present study provide an indication of how uncorrected astigmatism might influence a child's performance on more complex visual motor and perceptual tasks. The results of this study, in addition to results of a previous study of this population indicating that reading fluency is reduced in uncorrected astigmatic students but not in corrected astigmatic students [4], emphasize the benefits of providing full time correction for astigmatic children.

## Figures and Tables

**Table 1:** Comparison of demographics, vision, and refractive errors across three groups: students with low refractive error (control group), bilateral astigmats who completed the VMI and VMIp without correction, and bilateral astigmats who completed the VMI and VMIp with spectacle correction. Bolded  numbers represent acuity data included in secondary analyses controlling for acuity under VMI/VMIp test conditions.

	Control group: no/low refractive error	Bilateral astigmatism groups		*p* value
	Tested without correction *n* = 170	Tested without correction *n* = 88	Tested with correction *n* = 75	
Percentage female/male	46.5%/53.5%	47.7%/52.3%	50.7%/49.3%	=0.832

Mean age (SD, range)	11.50 (1.77, 8.21 to 15.87)	11.94 (1.77, 8.52 to 15.46)	11.53 (1.86, 8.35 to 15.47)	=0.150

Mean LogMAR (Snellen) uncorrected acuity (SD, range)	**−0.09 (20/16)** **(0.12, −0.30 to 0.50)**	**0.28 (20/38)** **(0.18, −0.10 to 0.70)**	0.25 (20/36) (0.17, −0.10 to 0.80)	<0.001^*∗*^

Mean LogMAR (Snellen) corrected acuity (SD, range)	−0.11 (20/16) (0.12, −0.30 to 0.70)	0.01 (20/20) (0.12, −0.20 to 0.30)	**0.00 (20/20)** **(0.10, −0.20 to 0.30)**	<0.001^*∗*^

Right eye astigmatism (SD, range)	0.25 (0.24, 0 to 0.75)	3.16 (1.26, 1.00 to 7.25)	3.07 (1.39, 1.00 to 6.75)	=0.649^†^

Left eye astigmatism (SD, range)	0.22 (0.22, 0 to 0.75)	3.24 (1.35, 1.00 to 7.50)	3.10 (1.36, 1.00 to 7.25)	=0.498^†^

Right eye spherical equivalent (SD, range)	+0.28 (0.50, −0.75 to +2.25)	+0.13 (2.00, −5.50 to +4.50)	−0.03 (2.00, −5.75 to +4.88)	=0.263

Left eye spherical equivalent (SD, range)	+0.30 (0.49, −0.88 to +2.25)	+0.15 (1.96, −5.25 to +4.75)	+0.07 (2.03, −6.13 to +5.00)	=0.455

^*∗*^Significance for one-way ANOVA across 3 groups. Post hoc analyses with Bonferroni correction: control group significantly differed from astigmatic groups (*p* values < 0.001). Astigmatic groups did not significantly differ.

^†^Significance for *t*-test comparison of two astigmatic groups. By definition, the control group has less astigmatism than the two astigmatism groups.
